# A prognostic model for gastric cancer based on histamine-associated prognostic genes

**DOI:** 10.1186/s41065-026-00659-w

**Published:** 2026-03-11

**Authors:** Yan Zhang, Na Liu, Xiao Dong, Yu Li, Jinrui Geng, Yonghong Li

**Affiliations:** 1 NHC Key Laboratory of Diagnosis and Therapy of Gastrointestinal Tumor, Gansu Provincial Hospital, No. 204, Donggang West Road, Chengguan District, Lanzhou, Gansu 730000 China; 2 Institute of Clinical Research and Translational Medicine, Gansu Provincial Hospital, No. 204, Donggang West Road, Chengguan District, Lanzhou, Gansu 730000 China; 3 Gansu University of Chinese Medicine, No. 35, Dingxi East Road, Chengguan District, Lanzhou, Gansu 730000 China

**Keywords:** Gastric cancer, Histamine, Prognostic genes, Prognostic model, Immune infiltration

## Abstract

**Background:**

Histamine has various associations with gastric cancer (GC); however, the mechanisms underlying histamine functions in GC have not been established. This study aimed to examine histamine-linked prognostic genes and mechanisms in GC.

Relevant data were sourced from public databases, and differential expression, multivariate Cox, univariate Cox, and machine learning regression analyses were performed to identify prognostic genes associated with histamine in GC. Subsequently, a prognostic model was constructed. Independent prognostic factors linked to GC prognosis were determined through regression analyses and used for nomogram model construction. Furthermore, Gene Set Enrichment Analysis (GSEA) and drug sensitivity and immune infiltration analyses were conducted to explore the potential function of these genes in GC from various perspectives. Finally, reverse transcription-quantitative polymerase chain reaction (RT-qPCR) was used to validate the expression of GRP, NPPB, SERPINE1, GAMT, MMRN1, and SLC22A16 in GC and paracarcinoma tissue.

**Results:**

GRP, NPPB, SERPINE1, GAMT, MMRN1, and SLC22A16 were identified as putative prognostic genes, and a prognostic model was constructed. Compared with the low-risk group (LRG), the survival rates in the high-risk group (HRG) were reduced. Moreover, only M, N, age, and risk scores could be used to construct the nomogram model, which could precisely predict the survival status of patients with GC. GSEA indicated that the development of GC may be associated with certain metabolic pathways. Furthermore, there were 23 distinct infiltrating immune cells between the HRG and LRG, including activated B cells. HRG and LRG showed remarkable variation in sensitivity to 100 drugs (e.g., AP.24534, pazopanib, and AZD8055). RT-qPCR revealed that GRP, NPPB, SERPINE1, GAMT, MMRN1, and SLC22A16 expression was markedly upregulated in GC tissues compared with paracarcinoma tissue.

**Conclusion:**

We identified six prognostic genes and constructed a prognostic model, which provides a theoretical basis for the relationship between histamine and GC as well as potential therapeutic targets for GC.

**Supplementary Information:**

The online version contains supplementary material available at 10.1186/s41065-026-00659-w.

## Background

Gastric cancer (GC) is one of the major invasive tumors prevalent worldwide, and its occurrence and mortality ranks among the highest in all malignancies [1]. Despite substantial advances in surgery, chemotherapy, targeted therapy, and immunotherapy, the prognosis for patients with advanced GC remains poor, with low 5-year survival rates [2]. This poor prognosis is largely attributable to GC’s insidious onset, low early-detection rate, and marked tumor heterogeneity. Therefore, identifying effective prognostic biomarkers and developing accurate predictive models is crucial for guiding individualized treatment strategies and improving patient outcomes [3].

Onset and progression of GC, like other solid tumors, is closely associated with immune regulation within the tumor microenvironment (TME) [4, 5]. Histamine is a classical biogenic amine and immune regulatory mediator that is involved in allergic and inflammatory responses and plays a complex and dual role in tumor biology [6]. By binding to its four G protein-coupled receptors (H1R–H4R), histamine can regulate immune cell function and attenuate multiple processes, including angiogenesis, cell proliferation, and apoptosis [7]. However, the precise mechanisms underlying the histamine-signaling pathway in GC and their association with prognosis have not been elucidated. Therefore, examining the prognostic significance of histamine-related genes in GC can provide unique insight into the tumor immune microenvironment as well as facilitate the identification of novel therapeutic targets.

To elucidate the role of histamine in GC, we used transcriptomic data from public databases to identify histamine-related prognostic genes in GC through multiple analytical approaches. A prognostic model was constructed based on these genes, and subsequently, various analytical methods were used to identify the mechanisms linking these prognostic genes to GC from various perspectives. This study provides a deeper understanding of the molecular mechanisms underlying GC and facilities the development of novel therapeutic strategies. Moreover, it holds promise for advancing innovative therapeutic approaches and personalized treatment for GC.

## Methods

### Data collection

TCGA-STAD (training set, accessed on February 6, 2025) gene expression, clinical, and survival data for GC were sourced from the TCGA database. The TCGA-STAD included 412 GC (385 specimens with survival information) and 36 control specimens; the GSE84426 (GPL6947, validation set) was mined from the GEO database. The GSE84426 consisted of 76 GC tissue samples with survival information. A total of 1,552 histamine-related genes (HisRGs) were compiled using the histamine-related pathways in the KEGG database, specific genes connected with histamine, and HisRGs in Hallmark, using “histamine” as the keyword (Supplementary Table 1) [8].

### TCGA-STAD dataset: Differential expression analysis

The “DESeq2” package (v 1.40.2) [9] was applied to the TCGA-STAD to acquire DEGs between GC and control specimens (adj. *p* < 0.05, |log_2_FC| > 1). Based on the adj. *p* values, results are presented using a volcano plot with the “ggplot2” package [10], with the top 10 gene names labeled. Furthermore, a heatmap generated by the “ComplexHeatmap” package shows the expression of the top 10 DEGs in a comparison of GC versus control samples [11].

### Recognition and function of candidate genes

DEGs and HisRGs were cross-analyzed using the “ggvenn” package (v 0.1.9) [12] to identify candidate genes. Subsequently, GO and KEGG analyses (*p* < 0.05) were performed using the “clusterProfiler” package (v 4.2.2) [13]. The top 10 results were selected based on *p*-value thresholds and visualized using the “ggplot2” package. Furthermore, the filtered candidate genes (interaction score ≥ 0.4) were imported into the String database to construct a protein–protein interaction (PPI) network. Finally, the interaction among the top 10 genes was visualized using Cytoscape software (v 3.8.2) [14].

### Identification of prognostic genes

To further identify prognostic genes, for the GC cases with survival outcomes of the TCGA-STAD, based on candidate genes, univariate Cox regression analysis (*p* < 0.05, HR ≠ 1) with PH assumption test (*p* > 0.05) using the “survival” package (v 3.7.0) [15], LASSO regression analysis via “glmnet” package (v 4.1-8) [16], and multivariate Cox regression analytics (*p* < 0.2) using the “survival” package were performed in sequence. Genes acquired from the final analysis step were designated prognostic genes.

### Construction of a prognostic model

To determine the value of the prognostic genes for the GC samples with survival information from TCGA-STAD, a risk score was computed for each GC sample using the following formula:

$$\text{risk score}=\sum\nolimits_{\mathrm{i}=1}^\mathrm{n}\mathrm{coef}\left(\mathrm{gene}_\mathrm{i}\right)\mathrm{expr}\left(\mathrm{gene}_\mathrm{i}\right)$$

Here, coef represents the risk parameter for each gene. Expr indicates the expression of each prognostic gene. Based on the optimum critical value of the risk score, GC samples were divided into an HRG and an LRG. The “survminer” package [17] was used to create Kaplan–Meier (K–M) curves for HRG and LRG, and the log-rank test was used to compare the survival disparities between the two groups (*p* < 0.05). Subsequently, the risk score curves, survival statuses, and levels of genes were visualized. Moreover, to analyze the veracity of the above model, the “timeROC” package (v 0.4) [18] was used to generate ROC curves representing 3, 5, and 7 years (AUC > 0.6). The same approach was used to validate the correctness of the prognostic model with GSE84426 data.

### Clinical feature analysis

To elucidate the bond between clinical features (N stage, T stage, gender, age, and M stage) and risk score for the GC samples with survival information of TCGA-STAD, a Wilcoxon test was applied to contrast risk scores for various clinical subgroups (*p* < 0.05). To acquire independent prognostic factors associated with prognosis, based on clinical features and risk score, a univariate Cox regression analysis (*p* < 0.05, HR ≠ 1) with PH assumption test (*p* > 0.05) and a multivariate Cox regression analysis (*p* < 0.05, HR ≠ 1) with a PH assumption test (*p* > 0.05) were performed in sequence using the “survival” package. In addition, for the GC samples with survival information from TCGA-STAD, the independent prognostic factors acquired from the multivariate Cox regression analysis were used to generate a nomogram model with the “rms” package (v 6.8-1) [19]. Grades were assigned to all independent prognostic factors, with total scores derived from the sum of the individual grades. With the increase in aggregate scores, a patient’s mortality rate increased. Finally, to evaluate the exactness of the nomogram for clinical prediction, calibration curves were drawn using the “regplot” package (v 1.1) [20] and ROC curves (AUC > 0.7) were drawn utilizing the “timeROC” package.

### Enrichment analysis

To identify pathways in the HRG and LRG in the GC specimens with survival metrics from TCGA-STAD, the “DESeq2” package was used to analyze distinctive expression between HRG and LRG, and the resulting log_2_FC was sorted in decreasing order. Subsequently, GSEA was achieved using “clusterProfiler” (*p* < 0.05, |NES| > 1, FDR < 0.25 [for controlling false positives caused by multiple testing]). Notably, “c2.cp.v7.2.symbols.gmt,” a reference gene set, was extracted from MSigDB. The top five pathways are displayed.

### Immune infiltration analysis

To evaluate the degree of infiltration by immune cells in the GC samples from the HRG and LRG from the GC specimens with survival information, the ssGSEA algorithm was applied along with the percolation abundance of 28 cells [21] between the two cohorts was quantified. The Wilcoxon test was used to compare the penetration difference of immune cells between the two cohorts (*p* < 0.05). The “psych” package [22] was used to determine the association between distinct immune cell types and prognostic genes (|cor| > 0.3, *p* < 0.05). The “estimate” package (v 1.0.13) [23] was used to calculate immune scores, ESTIMATE scores, and stromal scores for each GC sample. The scores between HRG and LRG were compared using the Wilcoxon test (*p* < 0.05).

### Drug sensitivity analysis

To probe the differences in sensitivity to standard chemotherapeutic drugs between the HRG and LRG, 138 drugs were acquired from the GDSC database. In the GC samples of TCGA-STAD, the “pRRophetic” package [24] was used to calculate the IC_50_ of drugs for each case. The Wilcoxon test was used to analyze the divergence in drug responsiveness between the two cohorts (*p* < 0.05). Based on *p* values, the IC_50_ values for the top 6 drugs with the most profound divergences between the LRG and HRG are presented.

### Collection and processing of samples

Ten GC tissue samples, along with corresponding paracarcinoma tissue specimens, were obtained from Gansu Provincial Hospital. This study was carried out according to the ethical guidelines provided by the World Medical Association Declaration of Helsinki. Prior ethical approval was obtained from the Ethics Committee of Gansu Provincial Hospital (2025 − 641). All patients provided informed consent. None of the tissue specimens were from patients who had undergone preoperative radiotherapy or chemotherapy. Immediately upon surgical excision, the samples were preserved at − 80℃ with an RNA stabilization solution.

### RT-qPCR assay

Total RNA was isolated from 10 matched GC and paracarcinoma tissues, which were snap-frozen in liquid nitrogen, using TRIzol reagent (Biomed, China). The RNA was diluted according to its concentration and reverse-transcribed into complementary DNA (cDNA) using a commercial reverse transcription kit (Biomed, China). Quantitative real-time PCR (qRT-PCR) was done on the AutoMolec 1600 instrument (Antobio, China). The thermocycling conditions were as follows: 180 s at 95 °C, followed by 40 cycles of denaturation for 15 s at 95 °C, then 60 s at 60 °C. The primer sequences are listed in Supplementary Table S2. Relative mRNA expression levels were determined using the 2^−ΔΔ Ct^ method, with GAPDH as the housekeeping gene for normalization. The Mann–Whitney U and Student’s t-tests were used to compare the two groups, with a *p*-value of < 0.05 considered statistically significant. Pearson’s correlation coefficient was used to assess the relationship between gene expression and risk score. The gene expression data were log2-transformed after adding one to each value.

### Statistical analysis

All analyses were performed using the R software (v 4.2.2). The Wilcoxon test was used to determine the differences between two groups (*p* < 0.05).

## Results

### Candidate gene functions in GC

**Fig. 1:**
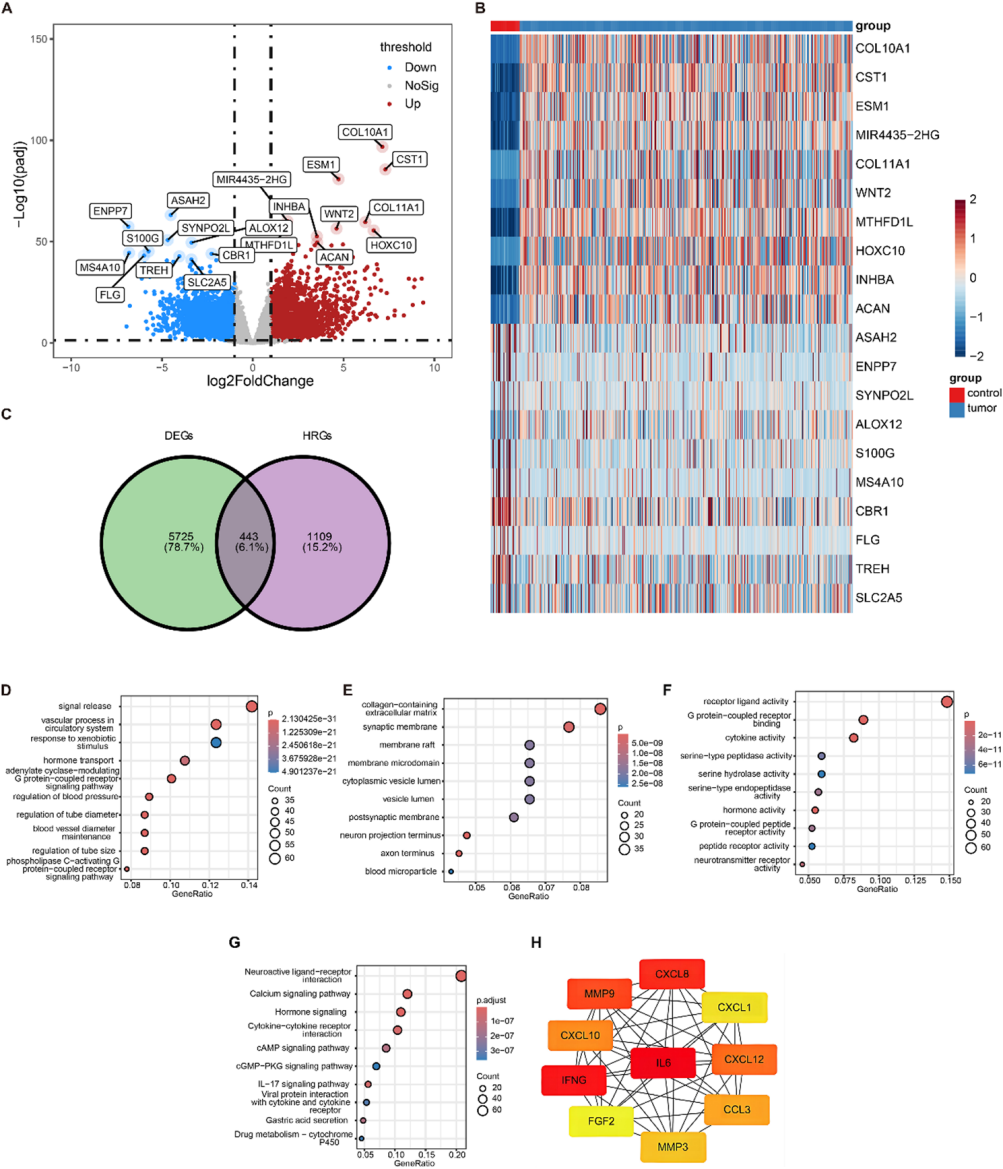
Identification and functional enrichment analysis of candidate genes. (**A**) A volcano plot showing the DEGs between tumor and normal samples. (**B**) The heat map indicates the top 10 upregulated and downregulated genes, with red representing high expression and blue representing low expression in tumor samples compared with normal samples. (**C**) Overlapping analysis of DEGs and HisRGs genes. (**D**) GO enrichment analysis of candidate genes in BP. (**E**) GO enrichment analysis of candidate genes in CC. (**F**) GO enrichment analysis of candidate genes in MF. (**G**) KEGG analysis of the candidate genes. The horizontal axis represents the number of genes enriched in GO entries as a proportion of all genes, and the vertical axis contains the name of the corresponding entry. The red and blue gradients indicate the *p*-value change. (**H**) Construction of a PPI network for the candidate genes. Rectangles represent genes, and gray bars indicate that the two genes interact

Our results indicated that compared with the control group, there were 6,168 DEGs in the GC group, which consisted of 3,359 upregulated DEGs and 2,809 downregulated DEGs. Based on adj. *p* values, all DEGs, and the top 10 upregulated and downregulated DEGs were displayed in a volcano plot (Fig. 1A). The heatmap also shows the expression profile of the top 10 up- and downregulated DEGs between the GC and control groups (Fig. 1B). The DEGs were intersected with the HisRGs, and 443 putative genes were identified (Fig. 1C). GO analytics revealed that the genes were primarily enriched in 2,419 functions (*p* < 0.05), which included 2,049 BP items (Fig. 1D), such as regulation of tube diameter, 115 CC items (Fig. 1E), including endoplasmic reticulum lumen, and 255 MF items (Fig. 1F), such as cytokine activity (Supplementary Table 3). A KEGG analysis revealed that these genes were primarily enriched in 105 pathways (*p* < 0.05), including environmental information processing (Fig. 1G, Supplementary Table 4). At the protein level, because IL6 has numerous interactions with other genes, indicates that IL6 may play an important role in GC (Fig. 1H).

### Utility of prognostic genes in prognostic models

**Fig. 2:**
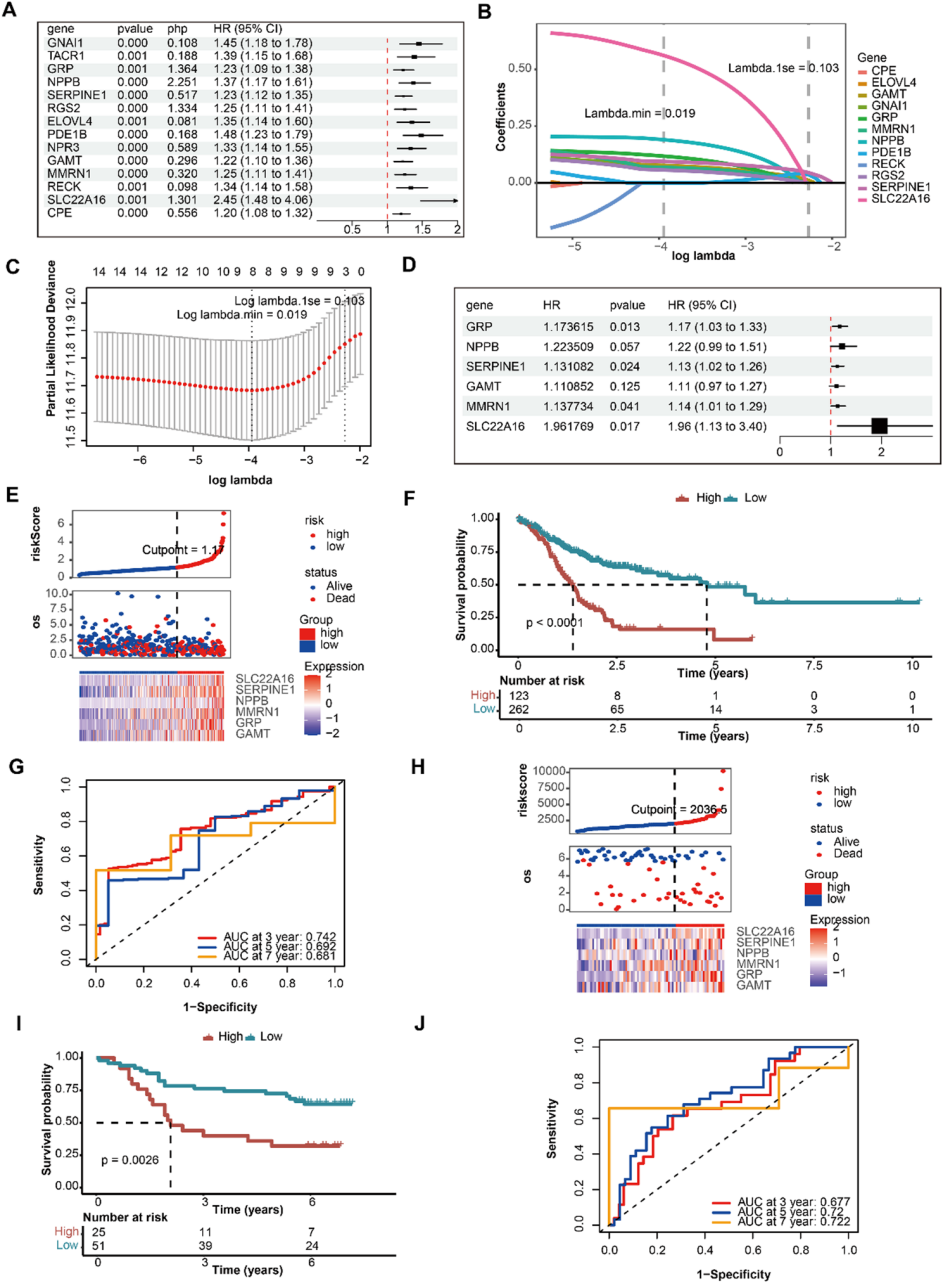
Prognostic gene screening and risk model construction. (** A**) Univariate Cox analysis of 14 candidate genes. (**B**) LASSO coefficient profile. (**C**) LASSO cross-validation curve. (**D**) Multivariate Cox analysis of six prognostic genes. (**E**) Risk score distribution, survival status, and gene expression patterns between the high-risk group (HRG) and low-risk group (LRG) using TCGA-STAD. (**F**) Kaplan–Meier (KM) survival analysis confirmed significantly poorer overall survival in HRG (*p* < 0.05). (**G**) Time-dependent ROC curves validating the model’s predictive accuracy (AUC > 0.6). (**H**) Validation of risk stratification using the GSE84426 cohort. (**I**) Consistent survival disadvantage observed in HRG (*p* = 0.0026). (**J**) ROC curves confirm the model’s robustness

The 14 genes were analyzed by univariate Cox regression (Fig. 2A). Notably, eight genes were generated by the LASSO algorithm (lambda.min = 0.019, corresponding to the minimum model error) (Fig. 2B, C). Moreover, six genes were identified by multivariate Cox regression (Fig. 2D). Thus, *GRP*, *NPPB*, *SERPINE1*, *GAMT*, *MMRN1*, and *SLC22A16* were considered prognostic genes for the development of a prognostic model. Relying on the optimal threshold value (1.17, minprop = 0.2) of the patient risk score, GC patients were divided into HRG (123 GC patients) and LRG (262 GC patients) in the TCGA-STAD (Fig. 2E). Moreover, survival of the patients in the HRG and LRG was also visualized (Fig. 2E). Compared with the LRG, the HRG showed a reduced survival rate (*p* < 0.0001) (Fig. 2F). The AUC was related to the ROC curves over 3-, 5-, and 7-year periods, with all exceeding 0.6. This indicates that the prognostic model was accurate (Fig. 2G). Notably, all prognostic genes were represented in the HRG with low representation in the LRG (Fig. 2E). Based on the median (2036.5) of the risk scores derived from the GSE84426 dataset, patients with GC were grouped into the HRG (25 GC patients) and LRG (51 GC patients) (Fig. 2H). In addition, the survival of GC patients was also examined (Fig. 2H). Survival outcomes were substantially poorer in the HRG compared with those in the LRG (*p* = 0.0026) (Fig. 2I). For ROC AUC values above 0.6, the risk model worked well (Fig. 2J). Expression of all prognostic genes was increased in the HRG, while being suppressed in the LRG (Fig. 2H). Results of the prognostic model using GSE84426 data indicated that it was accurate and reliable.

### Ability of independent prognostic factors to predict GC incidence

**Fig. 3:**
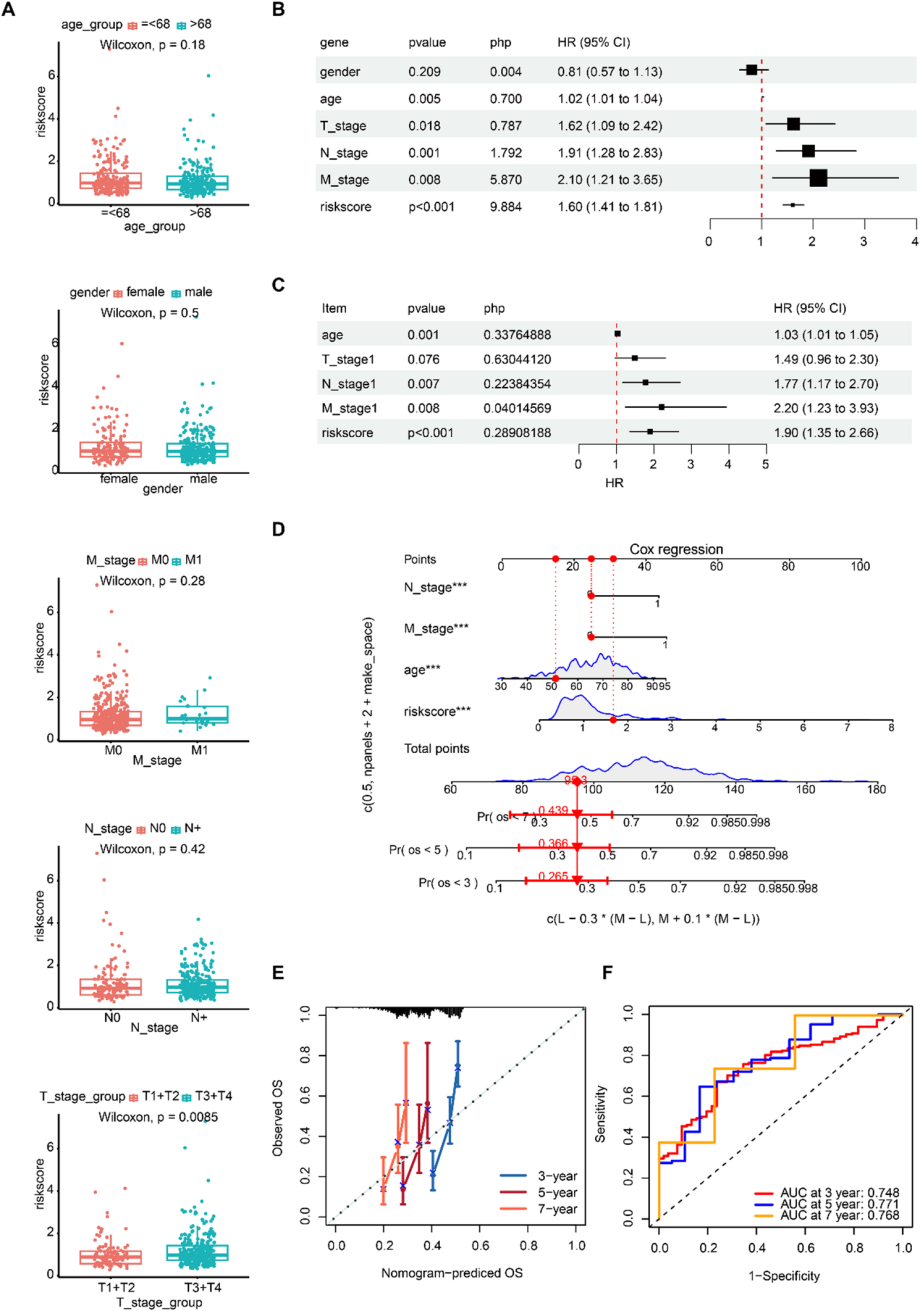
Nomogram model constructed using prognostic factors accurately predicts the mortality rate of GC patients. (**A**) The Wilcoxon test revealed a correlation between risk scores and age, gender, and TNM stage. (**B**) A forest plot based on the univariate regression analysis of age, gender, T, N, M, and risk scores. (**C**) A forest plot based on the multivariate regression analysis of age, gender, T, N, M, and risk scores. (**D**) Nomogram of independent prognostic factors. (**E**) Calibration curve of the nomogram model. (**F**) ROC curve of the nomogram model

Of these clinical features, only the risk scores between T1 + T2 and T3 + T4 exhibited considerable differences, whereas the risk scores of T3 + T4 were markedly higher (*p* = 0.0085) (Fig. 3A). Subsequently, univariate Cox regression analysis suggested that risk score, T stage, age, N stage, and M stage were associated with the prognosis of GC patients (*p* < 0.05, HR ≠ 1) (Fig. 3B). Only the N stage, age, M stage, and risk score could be used to build the nomogram model (Fig. 3C). The nomogram suggested that the independent prognostic factors forecast the mortality rate of patients, which surged with the increase in the aggregate points of the nomogram (Fig. 3D). The slopes of the standardization plots and the 3-, 5-, and 7-year periods were all bordering on 1, indicating the robustness of the line chart (Fig. 3E). Similarly, the AUC values were > 0.7, indicating the correctness of the line chart (Fig. 3F). Overall, the nomogram model constructed by prognostic factors accurately predicted the mortality rate of GC patients.

### Enrichment pathway and immune cells in GC

**Fig. 4:**
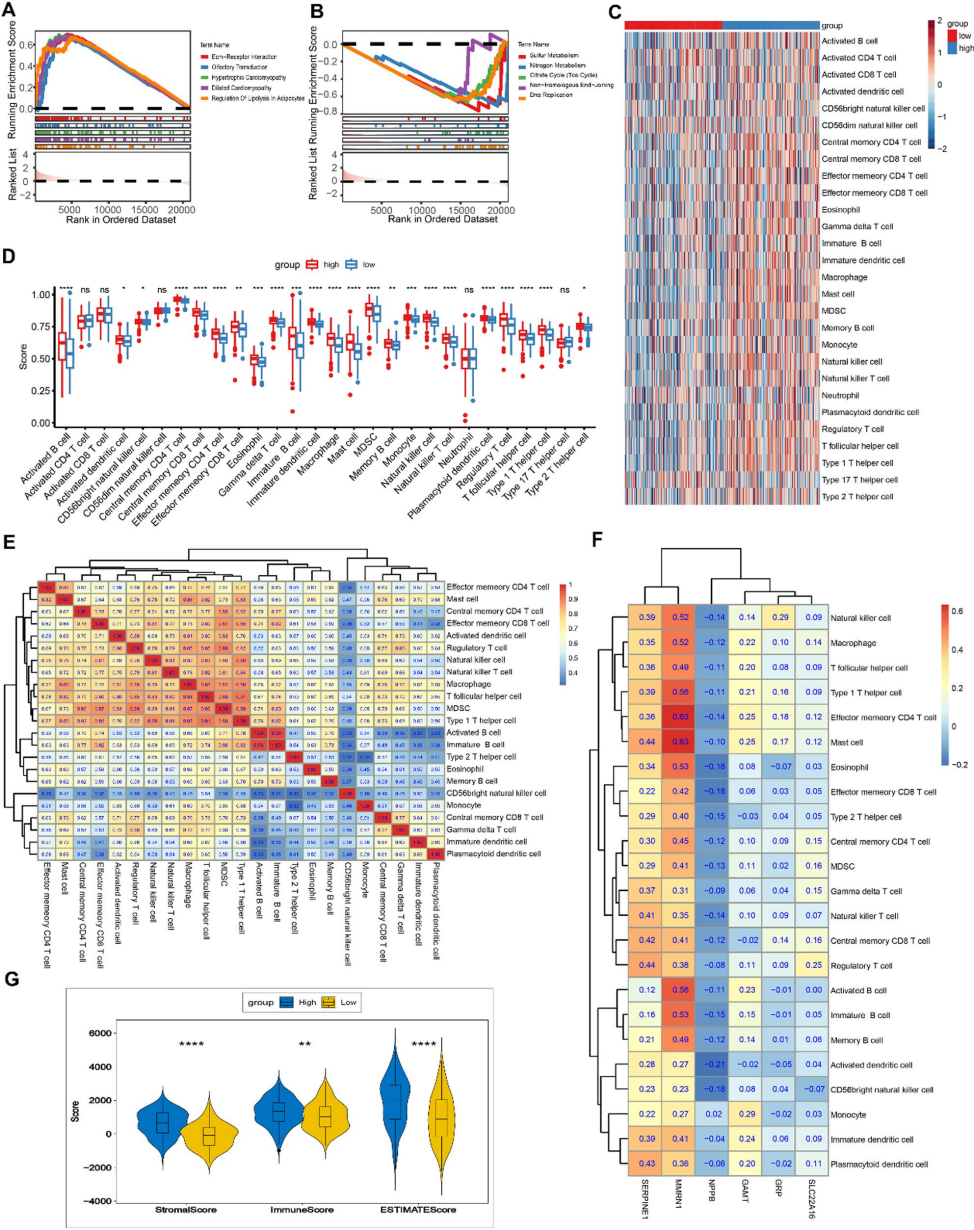
Functional and immune characteristics of the risk groups. (**A**) HRG was enriched in cytoskeleton remodeling and neuroactive ligand-receptor interactions. (**B**) LRG showed significant enrichment in metabolic pathways (e.g., TCA cycle). (**C**) Distinct immune cell infiltration patterns were observed between the HRG and LRGs. (**D**) Significant differences in 23 immune cell subsets were observed (e.g., mast cells and TEM cells). (**E**) Close intercellular communication networks among differentially infiltrated immune cells. (**F**) Prognostic genes showing strong correlations with immune infiltration (e.g., SERPINE1-mast cell: *r* = 0.63). (**G**) The HRG exhibited significantly higher stromal/immune scores, indicating an immunosuppressive microenvironment

A GSEA analysis revealed that the HRG was enriched in 71 pathways, and the LRG was enriched in 33 pathways (Supplementary Table 5). The HRG was highly enriched in olfactory transduction and dilated cardiomyopathy (Fig. 4A). The LRG was markedly enriched in sulfur metabolism and DNA replication (Fig. 4B). LRG may be associated with certain metabolic pathways. Moreover, the penetration abundance of 28 immune cells in the HRG and LRG was assessed (Fig. 4C). The degree of infiltration of 23 types of immune cells suggested considerable differences between the HRG and LRG (Fig. 4D). Moreover, the degree of infiltration of these immune cells was all lower in the LRG. Among the different immune cells, Type 1 T helper cells showed a positive correlation with T follicular helper cells (cor = 0.91, *p* < 0.0001) (Fig. 4E, Supplementary Table 6). MMRN1 had the highest positive correlation with mast cells (cor = 0.63, *p* < 0.0001) and effector memory CD4 T cells (cor = 0.63, *p* < 0.0001) (Fig. 4F, Supplementary Table 7). In addition, the immune scores (*p* < 0.01), ESTIMATE (*p* < 0.0001), and stromal scores (*p* < 0.0001) in the LRG were all inferior to those in the HRG (Fig. 4G). Taken together, the progression of GC may be associated with several immune pathways and various immune cells, such as mast cells.

### Variations in drug sensitivity

**Fig. 5:**
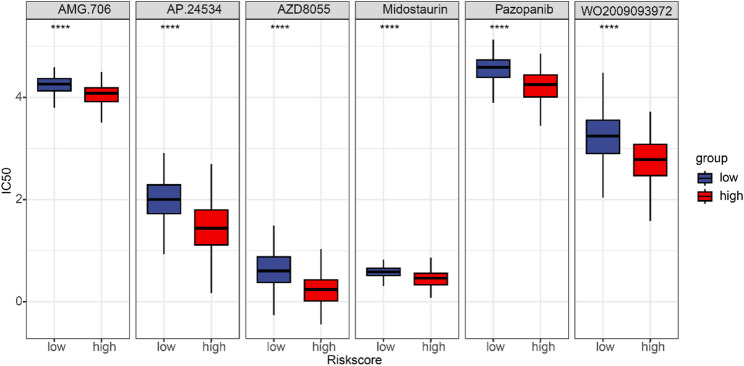
Correlation between the risk score and sensitivity to common chemotherapeutic drugs. Boxplot comparing the differences in IC50 scores for six drugs, including AP.24,534, pazopanib, AZD8055, midostaurin, WO2009093972, and AMG.706, between the high- and low-risk groups

Among 138 drugs examined, the HRG and LRG showed considerable differences in sensitivity to 100 drugs, such as A.770,041 and AG.014699 (Supplementary Table 8). The top six drugs with the smallest *p* values were as follows: AP.24,534, pazopanib, AZD8055, midostaurin, WO2009093972, and AMG.706 (*p* < 0.0001) (Fig. 5). Of these six drugs, the IC_50_ values in the HRG were markedly lower compared with those in the LRG. Thus, the HRG was more sensitive to drugs, such as AP.24,534.

### Expression validation of six histamine-related prognostic genes in GC

**Fig. 6:**
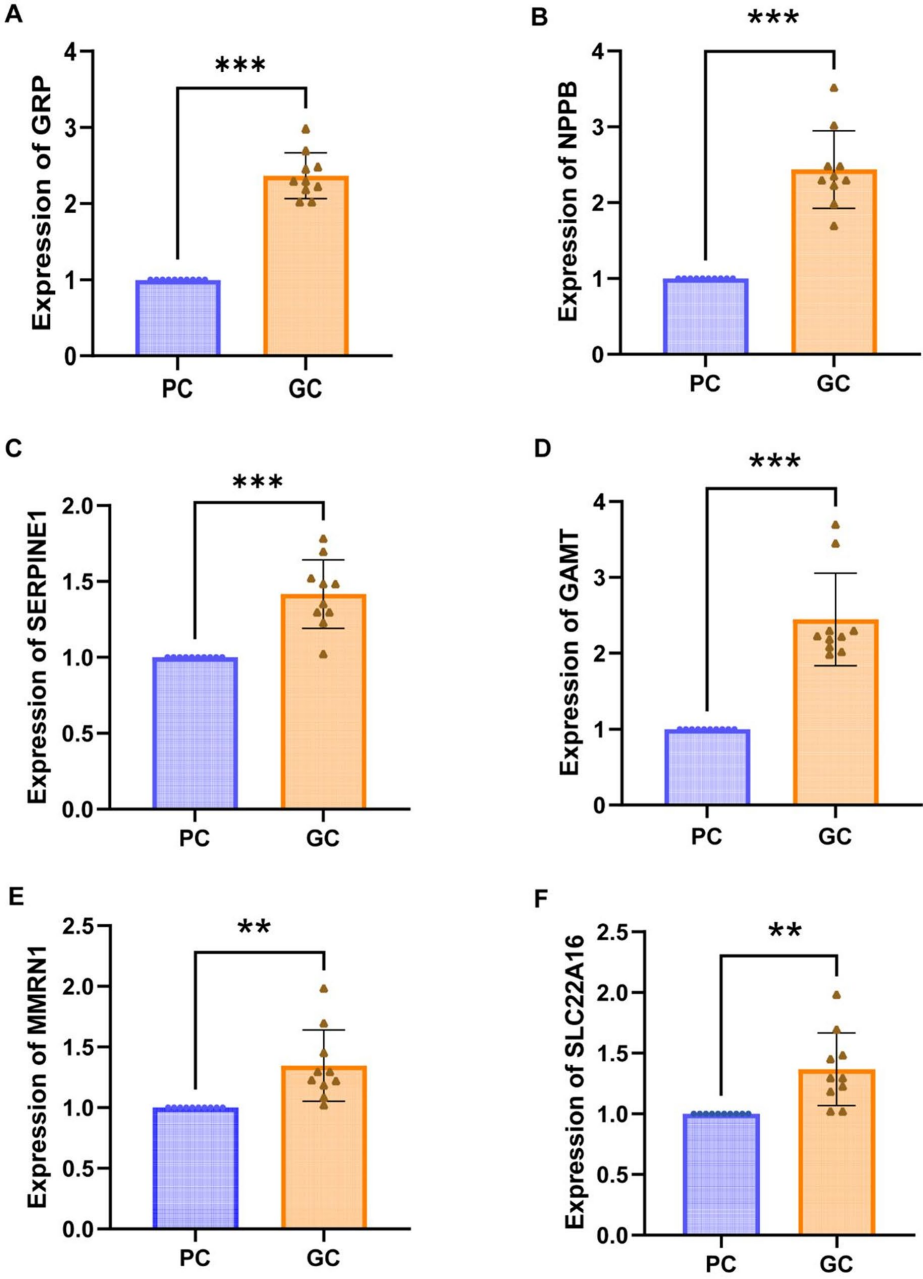
Validation of prognostic gene expression levels by qRT-PCR. (**A**) *GRP*. (**B**) *NPPB*. (**C**) *SERPINE1*. (**D**) *GAMT*. (**E**) *MMRN1*. (**F**) *SLC22A16*. (*p < 0.05; **p < 0.01; ***p < 0.001)

The expression of six histamine-related prognostic genes (*GRP*, *NPPB*, *SERPINE1*, *GAMT*, *MMRN1*, and *SLC22A16*) was measured in GC and para-cancerous tissue (PC). The results indicated that the expression of *GRP*, *NPPB*, *SERPINE1*, *GAMT*, *MMRN1*, and *SLC22A16* was significantly higher in GC tissues compared with PC tissues (Fig. 6).

## Discussion

GC is one of the leading causes of cancer-related deaths globally. It is characterized by a complex pathogenesis and substantial regional heterogeneity. This high heterogeneity and intricate TME of GC make the development of precise prognostic tools imperative. Leveraging transcriptomic data from public databases, we screened histamine-related prognostic genes in GC using multiple analytical approaches. A prognostic model was subsequently constructed based on these genes, followed by a comprehensive analysis of the molecular processes linking these genes to GC through a range of bioinformatic methods from multiple perspectives.

By integrating differential expression analysis, CORE regression, LASSO algorithm, and various machine learning methods, we identified six histamine-related prognostic genes (*GRP*, *NPPB*, *SERPINE1*, *GAMT*, *MMRN1*, and *SLC22A16*) in GC. All six genes were significantly overexpressed in GC tissues compared with normal tissues. Moreover, they were markedly higher in the HRG compared with those in the LRG. Their high expression was consistently linked to poor prognosis. This synergistic overexpression pattern suggests that these genes play a carcinogenic role in GC development, emphasizing the potential clinical value of histamine-related signaling pathways in GC progression. GRP encodes gastrin-releasing peptide, a neuropeptide that regulates gastrin secretion and gastrointestinal function by binding to the GRPR receptor [25]. The GRP–GRPR axis promotes tumor growth in various malignant tumors through autocrine stimulation and the release of proinflammatory cytokines [26]. Furthermore, we observed that high GRP expression is associated with poor prognosis in GC, consistent with its known proproliferative and proinflammatory effects, suggesting that targeting GRP signaling has therapeutic potential.

*NPPB* encodes brain natriuretic peptide (BNP), which is considered a cardiac hormone regulating cardiovascular homeostasis [27]. However, recent studies suggest that BNP may be involved in tumor biological processes, such as the correlation between mediastinal involvement and serum NT-proBNP levels [28]. In this study, high *NPPB* expression predicted poor prognosis in GC, which suggests a possible interaction between the cardiovascular system and tumors. However, the underlying mechanisms warrant further study, especially because of the increasing recognition of metabolic and hormonal factors as key regulators of the TME. Immune infiltration analysis further revealed a moderate correlation between *NPPB* expression and various immune cells. This suggests its potential involvement in the recruitment or functional regulation of immune cells within the gastric TME.

SERPINE1 (PAI-1) is a serine protease inhibitor that is an important regulator of the fibrinolytic system [29]. It was significantly overexpressed in high-risk patients in the present study. It promotes angiogenesis, inhibits apoptosis, and induces tumor migration and metastasis [30]. Immune infiltration analysis revealed a significant positive correlation between SERPINE1 expression and mast cell infiltration (*r* = 0.63, *p* < 0.0001), which is consistent with its role in TME remodeling. This result is significant, as mast cells were significantly enriched in the HRG, which suggests that SERPINE1 participates in shaping the immunosuppressive microenvironment of GC.

GAMT catalyzes the final step of creatine synthesis [31]. It promotes the progression of clear cell renal cell carcinoma by inhibiting p53 [32]. In the present study, high GAMT expression predicted a poor prognosis in GC, suggesting that metabolic reprogramming (especially creatine metabolism) plays an important role in GC development. GSEA analysis further supports this view. The LRG was enriched in metabolic pathways, such as sulfur metabolism, nitrogen metabolism, and the TCA cycle, whereas the HRG was enriched in cytoskeleton remodeling and neuroactive ligand-receptor interaction pathways, which suggests that high-risk patients exhibit metabolic characteristics more conducive to tumor growth.

MMRN1 is mainly involved in hemostasis [33] and is a prognostic biomarker for various cancers [34]. It is a risk factor in the GC microenvironment [35], which is consistent with our findings. Immune-related analyses revealed that MMRN1 was positively associated with mast cells and effector memory CD4⁺ T cells (both *r* = 0.63, *p* < 0.0001), suggesting its potential involvement in immune cell recruitment and activation. Differences in the infiltration of 23 immune cells were observed between the HRG and LRG, further indicating a regulatory role of these HisRGs in shaping the GC immune microenvironment.

*SLC22A16* encodes a carnitine transporter that plays an important role in fatty acid metabolism and energy homeostasis. Although it may be associated with chemosensitivity by mediating platinum-based drug uptake [36], we found that its high expression was associated with poor prognosis in GC. This seemingly contradictory result may stem from the dual nature of *SLC22A16* function. Although it promotes drug influx, its dominant lipid metabolism function may provide a survival advantage for cancer cells during metabolic stress.

Overall, we constructed a robust histamine-related prognostic marker system for GC, which revealed that high expression of *GRP*, *NPPB*, *SERPINE1*, *GAMT*, *MMRN1*, and *SLC22A16* can define a high-risk subtype of GC with metabolic abnormalities and an immunosuppressive microenvironment. These results not only enhance our understanding of the role of histamine in GC development, but also provide a theoretical basis and translational foundation for patient risk stratification and individualized treatment strategies.

After characterizing the individual roles of these prognostic genes, we examined their broader function by performing pathway enrichment analyses to identify the key signaling networks and metabolic processes that distinguish HRG from LRG GC patients. GO and KEGG analyses were conducted using the R package clusterProfiler, and the patient samples were divided into HRG and LRG based on their risk scores. HRG was significantly enriched in pathways, such as Cytoskeleton in Muscle Cells, Neuroactive Ligand-Receptor Interaction, and Hormone Signaling, which are commonly associated with the malignant progression of tumors. The dynamic reorganization of the cytoskeleton, particularly the actin cytoskeleton, plays an important role in the epithelial–mesenchymal transition and the acquisition of invasive ability in tumor cells. For example, filamin A, an actin-crosslinking protein, connects the cell membrane to the cytoskeleton and is involved in signal transduction and the maintenance of cell morphology. Its expression is upregulated in GC tissues, where it mediates Rac1 activation in GC cells through RhoGDI2 [37]. In mechanistic studies of GC, GNG7 is co-expressed with several genes in the neuroactive ligand-receptor interaction pathway, such as SST, NPY, and GHRL. The expression of the GNG7 gene is downregulated in GC, which may have a tumor-suppressive effect by regulating multiple genes in the neuroactive ligand-receptor interaction pathway [38]. Hormone signaling pathways primarily involve the actions of sex hormones, such as estrogen, progesterone, and androgens, through their receptors inside and outside of the cell. These signaling pathways play important roles in the onset, progression, prognosis, and gender differences in GC. The expression of estrogen receptor beta (ERβ) is significantly lower in GC tissues compared with the normal gastric mucosa, suggesting that it may have a suppressive role in GC development [39].

Conversely, the LRG exhibited significant enrichment in metabolic pathways, including sulfur metabolism, nitrogen metabolism, and the citrate cycle (TCA cycle). The transsulfuration pathway is a core pathway in sulfur metabolism. Key enzymes in this pathway, such as cystathionine β-synthase (CBS), are upregulated in GC cells. This promotes tumor cell survival and drug resistance by modulating redox status and inhibiting ferroptosis [40]. In nitrogen metabolism, alterations in urea cycle enzyme expression may result in nitrogen accumulation or redistribution, thereby supporting the increased metabolic demands of tumor cells [41, 42].

Although these metabolic alterations provide critical insights into tumor biology, the TME, particularly its immune component, plays an important role in determining patient outcomes. Because our risk model successfully stratified patients based on HisRGs and considering histamine’s well-established immunomodulatory functions, we examined the immune landscape differences between risk groups. Immunotherapy is a promising treatment for various cancers, such as lung adenocarcinoma [4, 43]. We determined the correlation between the new risk model and the tumor immune microenvironment in GC and identified 23 differentially expressed immune cells between the HRG and LRG. Notably, Tfh cells, mast cells, and effector memory CD4 T cells exhibited significant differences between the HRG and LRG. We found that the percentage of Tfh cells (Tfh%) in GC patients was significantly increased, and the expansion of Tfh cells contributed to immune suppression in GC [44]. Another study found that by digesting gastric mucosal tissue into a single-cell suspension to measure CD4 + T cells in the stomach, an increase in mucosal CD4 + T cells was evident following *Helicobacter pylori* infection. This supports the potential role of CD4 + T cells, including TEM cells, in immunotherapy [45]. Furthermore, previous studies demonstrated that tumor-infiltrating mast cells promote GC growth by stimulating ICOS+ regulatory T cells through the IL-33/IL-2 axis [46].

The distinct immune microenvironment profiles observed between HRG and LRG suggest fundamental biological differences that may extend beyond immune characteristics alone, which may be attributed to the activation of specific signaling pathways, including porin activity and G2/M DNA replication checkpoint [47]. These differences may influence therapeutic responses, specifically for chemotherapeutic agents. Therefore, we determined whether our risk stratification could predict differential drug sensitivities, which would have substantial implications in personalized treatments. We adopted the R package “pRRophetic” to calculate the IC₅₀ values for the STAD patient samples in the training set. The IC_50_ differences between the HRG and LRG for each drug were compared using the Wilcoxon test. The results indicated that 100 chemotherapy drugs showed significant differences between the HRG and LRG. For example, pazopanib combined with 5-FU and oxaliplatin has been used as first-line treatment for advanced GC [48], and axitinib, either alone or in combination with chemotherapy drugs, has shown antitumor activity in vitro and in vivo against human GC cells [49, 50].

## Conclusions

In summary, our histamine-related gene signature provides a robust framework for risk stratification and personalized treatment selection in GC patients; however, this study had several limitations. First, the retrospective nature of the analysis, relying on publicly available datasets (TCGA and GEO), may introduce selection bias and unmeasured confounding factors. Second, despite efforts to harmonize the data, inherent heterogeneity (e.g., age, geographical distribution) and potential batch effects (e.g., data collection times, experimental technical platforms, or operational procedures) across different datasets may affect the robustness of the prognostic model. Moreover, although we validated our model in an independent cohort (GSE84426), further multicenter prospective studies with standardized protocols are needed to confirm its generalizability. Third, the sample size, particularly for subgroup analyses (e.g., based on clinical stages), was limited, which may have affected the statistical power to detect subtle associations. Finally, although we examined immune infiltration and drug sensitivity, the model’s biological mechanisms require experimental validation to establish causality.

Although the immune microenvironment analysis revealed a correlation between risk scores and immune cells, studies of the underlying mechanisms, such as cytokines and immune checkpoints, are still preliminary and require further investigation. In the drug sensitivity analysis, although the HRG showed lower sensitivity to paclitaxel, further validation is needed to rule out potential confounding factors. Future studies should further refine the relationship between the immune microenvironment and the prognostic model based on larger sample sizes, while integrating new biomarkers, such as liquid biopsy, to enhance the model’s accuracy. Moreover, combining this model with drug response prediction to support personalized treatment, specifically in the context of immunotherapy and targeted therapy, may improve treatment outcomes and prognosis for GC patients. 

## Supplementary Information

Supplementary Material 1.

## Data Availability

The datasets used in this study were obtained from The Cancer Genome Atlas (TCGA) (https://portal.gdc.cancer.gov/) and Gene Expression Omnibus (GEO) (https://www.ncbi.nlm.nih.gov/geo/query/acc.cgi?acc=GSE84426).

## Abbreviations

BNP: Brain natriuretic peptide
GC: Gastric cancer
GSEA: Gene Set Enrichment Analysis
HisRG: Histamine-related genes
HRG: High-risk group
LRG: Low-risk group
PC: Para-cancerous
